# Supplementary figures and images for: A New Pathogenic Variant in POU3F4 Causing Deafness Due to an Incomplete Partition of the Cochlea Paved the Way for Innovative Surgery
Source: Genes (Basel). 2021 Apr 21;12(5):613. doi: 10.3390/genes12050613 (PMC8143104; doi:10.3390/genes12050613)

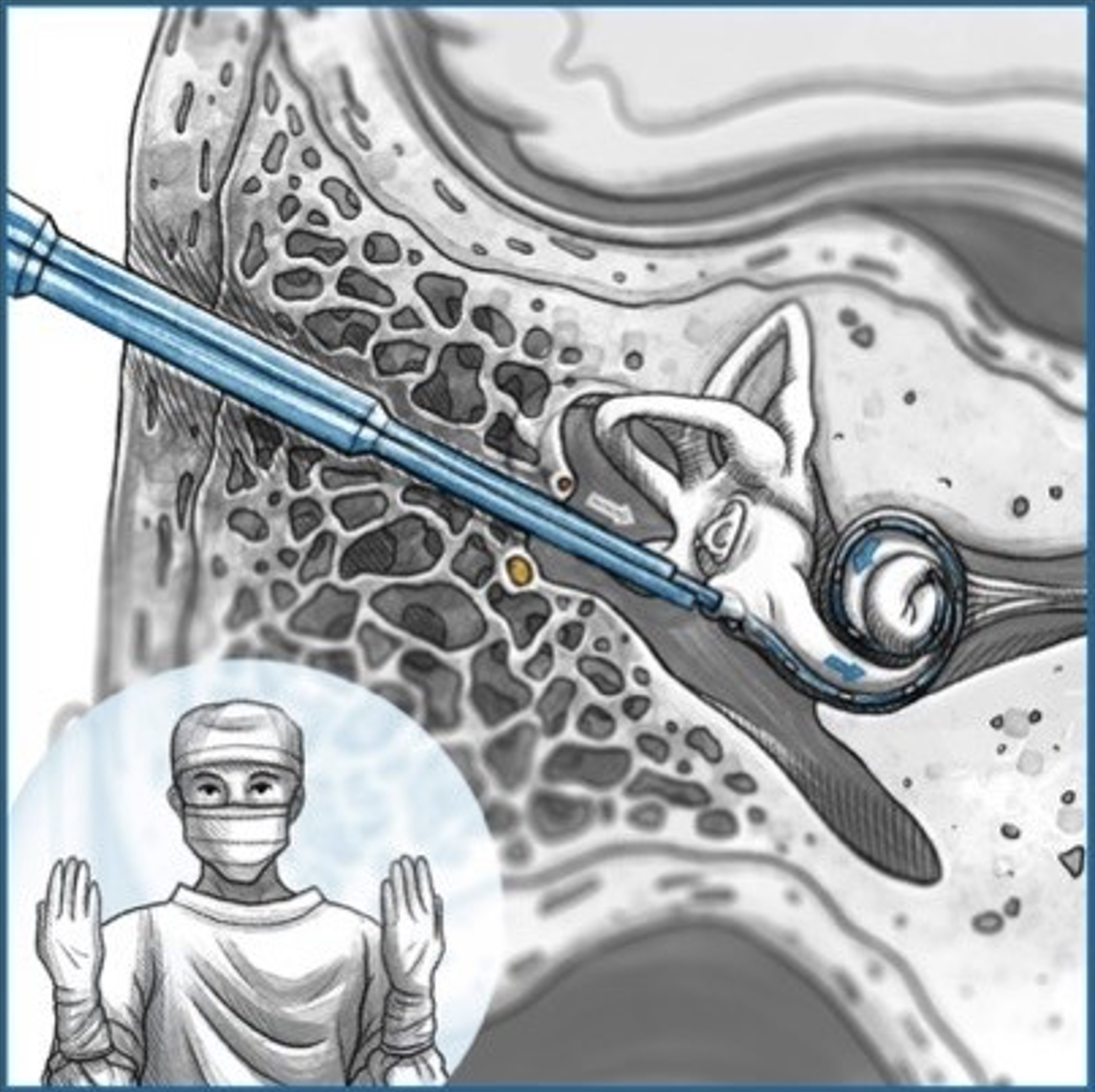

Supplement: Supplementary file 1 [file genes-12-00613-s001.zip › Supplementary/figure S2.png]

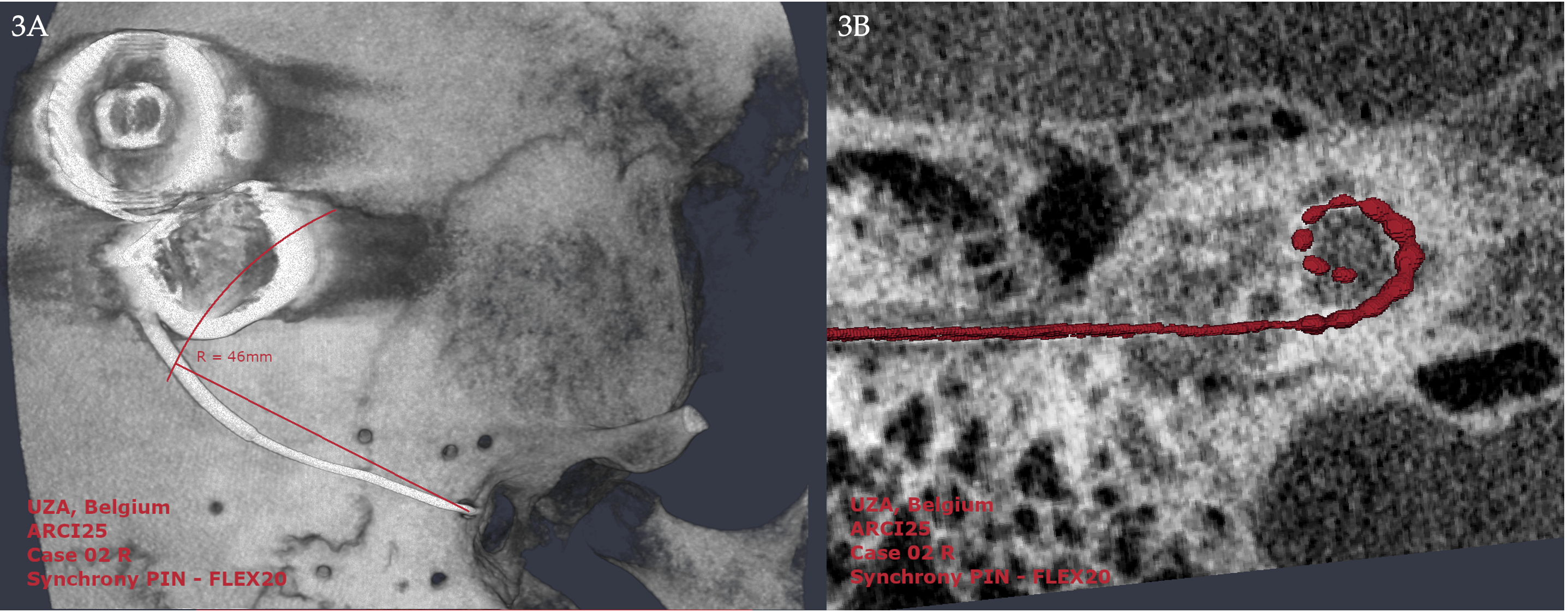

Supplement: Supplementary file 1 [file genes-12-00613-s001.zip › Supplementary/Figure S3.png]

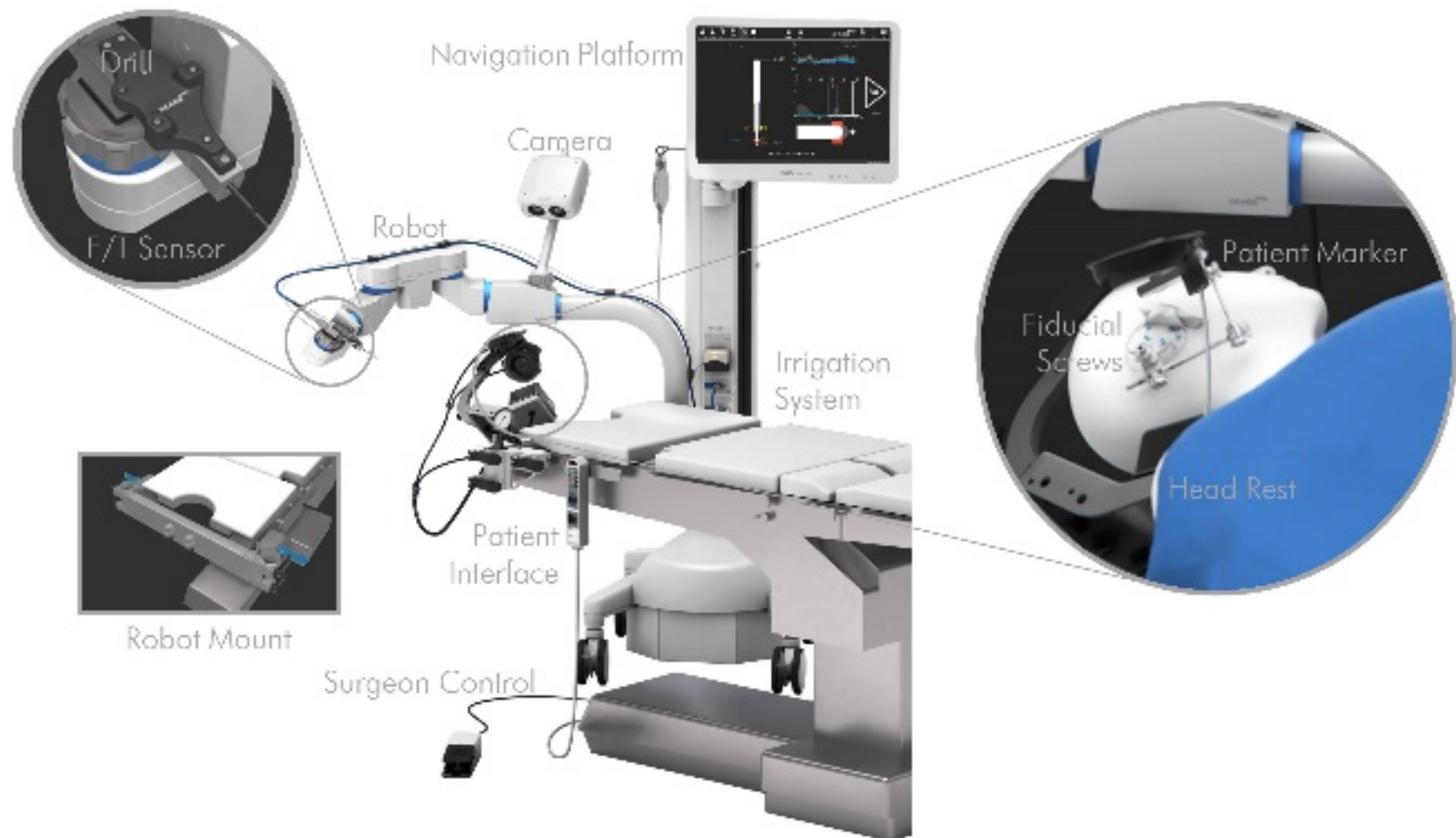

Supplement: Supplementary file 1 [file genes-12-00613-s001.zip › Supplementary/Figure S1.pdf]
